# canSAR: updated cancer research and drug discovery knowledgebase

**DOI:** 10.1093/nar/gkt1182

**Published:** 2013-12-03

**Authors:** Krishna C. Bulusu, Joseph E. Tym, Elizabeth A. Coker, Amanda C. Schierz, Bissan Al-Lazikani

**Affiliations:** Cancer Research UK Cancer Therapeutics Unit, The Institute of Cancer Research, London, SM2 5NG, UK

## Abstract

canSAR (http://cansar.icr.ac.uk) is a public integrative cancer-focused knowledgebase for the support of cancer translational research and drug discovery. Through the integration of biological, pharmacological, chemical, structural biology and protein network data, it provides a single information portal to answer complex multidisciplinary questions including—among many others—what is known about a protein, in which cancers is it expressed or mutated, and what chemical tools and cell line models can be used to experimentally probe its activity? What is known about a drug, its cellular sensitivity profile and what proteins is it known to bind that may explain unusual bioactivity? Here we describe major enhancements to canSAR including new data, improved search and browsing capabilities and new target, cancer cell line, protein family and 3D structure summaries and tools.

The translational research and drug discovery communities are still grappling with the mixed blessing of the deluge of data that has been the result of international coordinated efforts including the International Cancer Genome Consortium (1), the International Molecular Exchange (IMEx) protein interaction data consortium (2) and cellular drug profiling (3,4), and increasingly powerful sequencing and screening technologies. Rapidly accelerating data growth is spanning the fields of genomics (1,5,6), chemical biology (4), structural biology (7) and systems biology (2). Many powerful resources now exist to allow users to interrogate these data, e.g. (8,9). While some integrate several data types such as gene expression with compound activities (10,11) or different types of omics data (8), these resources typically focus their strengths on particular core areas such as genomics (8), drug action (9,12) or expression correlations (10).

canSAR, initially described in 2011 (13), was the first—and to our knowledge, remains the only—resource to integrate disparate and multidisciplinary data spanning biology, pharmacology, chemistry, structural biology and protein networks. Importantly, it was designed not merely to link and cross-reference these scientific domains, but to enable researchers to reach key information rapidly and answer multidisciplinary questions regardless of their background or areas of expertise. Since it was initially launched, canSAR has been visited by >32 000 users from >100 countries. The user-base includes biologists, chemists, structural biologists and clinicians. Here we describe major updates to canSAR data and its functionality.

## DATA CONTENT AND GROWTH

canSAR contains the full complement of the human proteome as well as 16 332 proteins from 2136 model organisms that fall into 8631 major protein families. It contains annotations and data for >11 000 cell lines, both cancer and nontransformed cell lines, and 3690 patient-derived tissue samples with >10 000 experimental result sets (for breakdown see http://cansar.icr.ac.uk/cansar/data-sources/). The fully searchable 2D structure and annotation for nearly one million small molecule drugs and chemical probes have collectively >8 000 000 experimental bioactivities as well as 10 million calculated properties. There are >93 000 3D structures for 31 130 proteins, collectively containing 16 700 ligands determined in complex with a protein. We have annotated and classified these ligands into different classes as described below and identified ca 16 000 ligands that are functionally relevant. All ligands have 3D ligand-interaction maps in canSAR. In addition to the collated and annotated data, canSAR contains curation and additional analyses including 4544 3D-structure–based, 8197 Ligand-based and 9446 protein-network–based druggability results for human proteins. Importantly, all data from all areas of research are seamlessly integrated and are fully referenced to their original sources and specific publications where available to ensure that researchers can rapidly identify the original source of the information without complex and lengthy searching.

## NEW SEARCH AND BROWSING CAPABILITIES

**Figure 1.**
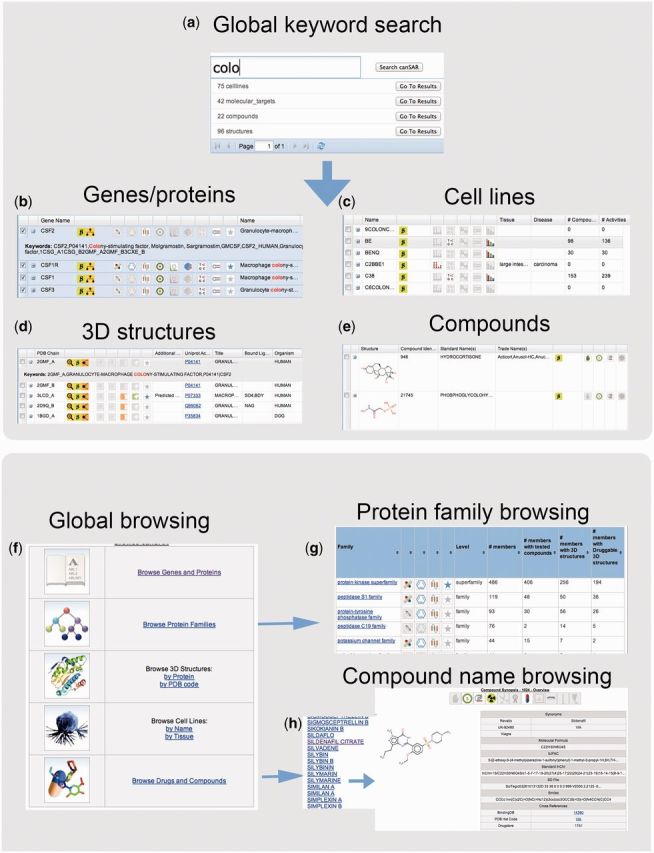
Global keyword searching: (**a**) the single global search capability that enables keyword searches to be performed across (**b**) genes and proteins, (**c**) cell lines (**d**) 3D structures and (**e**) compounds. The results are displayed in tabular forms with icons representing the availability of data such as cancer mutations and bioactive chemical probes. The user can sort the results based on these data. (**f**) New browsing functionality that allows exploring canSAR’s content through browsing genes and proteins, protein families, 3D structures or drugs and compounds. For example, (**g**) protein families have a summary feature where the user can sort and select families based on the types of data available. (**h**) Molecular targets, compounds and cell lines may be browsed by name.

canSAR now has a single global search capability (Figure 1a) that enables keyword searches to be performed across the system, thus speeding up the retrieval of data. Additional, object-specific search capabilities such as protein sequence and chemical structure searches remain in place.

Browsing canSAR data can now be accomplished through protein/gene name alphabetical dictionaries, protein-families, 3D structure and compound dictionaries (Figure 1f). These new search and browsing capabilities allow users to explore the data in canSAR in a broader way, e.g. the user can rank members of a particular protein family based on the availability of reported cancer mutations, bioactive chemical probes, druggability or can browse through all cell lines from a particular tissue type.

## TARGET SYNOPSES

**Figure 2.**
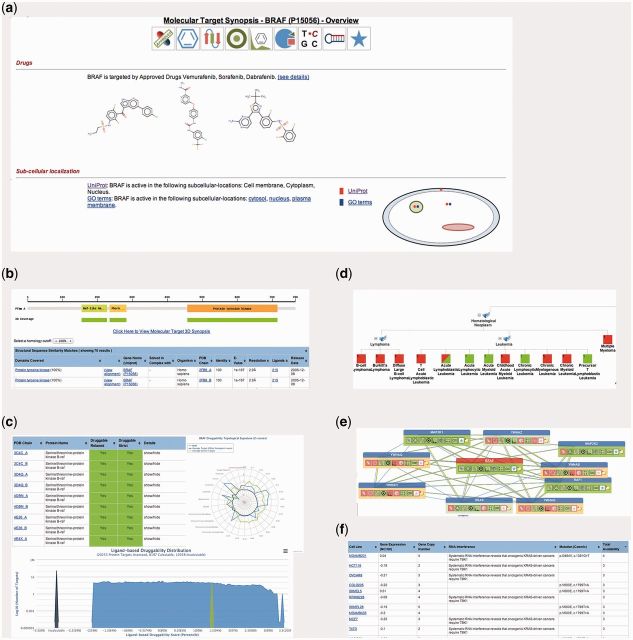
(**a**) The molecular target wiki page provides a complete, human readable, summary of the data stored for a particular protein and contains links to more in-depth data. The header icons indicate the types of information that are available, such as mutations, 3D structures and bioactivities as well as showing if the protein is an approved drug target. Detailed information such as drugs, (**b**) domain and protein 3D-structure availability, (**c**) druggability prediction using three alternative approaches as well as (**d**) tumor-tissue and cell line gene expression and mutation can be explored in detail and linked through to original sources. (**e**) Protein interaction networks are annotated with the chemogenomic data summaries from canSAR enabling exploration of deregulated and/or druggable network members. (**f**) Cell line data matrix provides a single view on mutation, expression, copy number variation and RNAi studies, where available, in one tabular summary.

One of the most popular uses of canSAR is retrieving instant summaries about a particular target of interest. To enhance this ability, we have developed a new ‘wiki’-style summary page (Figure 2a) that distils the key information about the target in a human-readable summary form and covers function, cellular localization, drugs and clinical candidates, cancer mutations, RNAi data, expression levels in cancer and nontransformed cell lines and drug or chemical probe bioactivity information among others. Each section is linkable and explorable with drill-down capabilities. The cell-line matrix displays all available information for the specific target in all cell lines, so in one table, the user can see the mutation state, expression level, and, where it exists, RNAi information (Figure 2f). The interactive protein-interaction networks provide a powerful resource that not only identifies protein interactions of the target of interest, but also provides chemical biology annotation and genomic information in an ‘at-a-glance’ form so that the user can immediately identify any drug targets in the network, or targets with known bioactive chemical probes, that are RNAi screen hits or that have known mutations in cancer (Figure 2e). Each protein in the network is colored according to its chemical tractability, thus highlighting proteins within the immediate network of the target of interest that would be most amenable to drug discovery.

## CELL-LINE SYNOPSES

**Figure 3.**
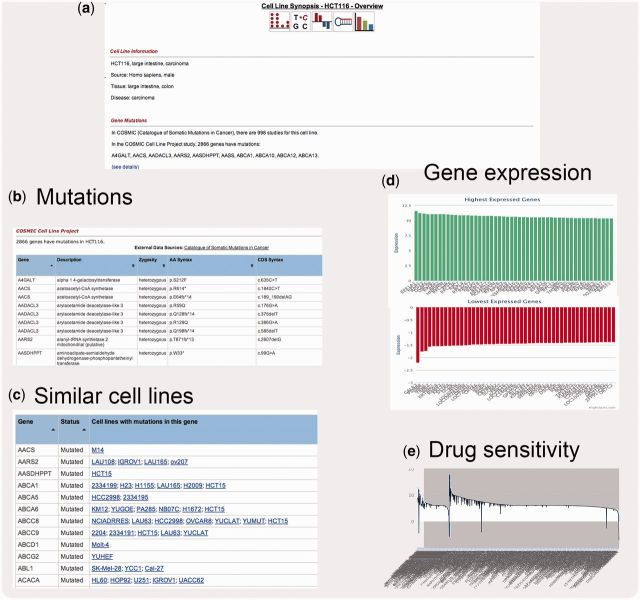
The new Cell Line synopsis summarizes the data stored about particular cell lines. (**a**) The overview page presents the highlights including a banner of icons indicating availability and status of different data such as mutations, bioactivities, RNAi and gene expression. All data can be explored in detail including (**b**) reported mutations, (**c**) listing of ‘genetically’ similar cell lines based on mutational status, (**d**) highest and lowest expressed genes and (**e**) drug sensitivity profiles compiled from different sources.

We have developed a synopsis page for each cell line in canSAR (Figure 3a) covering cancer and nontransformed cell lines. These cell-line synopses summarize key information about a cell line including its tissue of origins, key mutations and gene copy-number variations, the highest and lowest expressing genes, the number and the activity of drugs or chemical probes tested against it, among other descriptors. In addition, detail is provided including full mutation reports and comparison with other cell lines (Figure 3) to enable rapid identification of genetically similar or complementary cells, drug/chemical probes sensitivity profiles and RNAi experimental data summaries.

## PROTEIN FAMILY SYNOPSES

**Figure 4.**
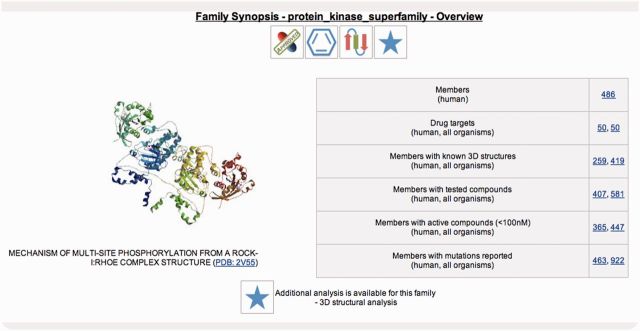
The Protein Family synopsis summarizes the data held about a particular family, including the number of druggable members, the number of members that have bioactive compounds and those members that are known to have mutations in cancer. These are linked directly to the full chemical bioactivity data and their publications and full mutation data as well as other annotation. All subfamilies and individual proteins can be reached through an interactive family tree.

It is often useful to view the information around a target family as opposed to individual targets. This can be the case when multiple family members are involved in the same biological process or when, say in a drug discovery context, the chemical modulation of certain family members needs to be avoided to prevent adverse reactions. To enable the full chemogenomic exploration of protein families, we have developed protein family synopsis pages (Figure 4) that summarize the number of family members, the number of members that are druggable, that have bioactive compounds and that are known to have mutations in cancer. The family definitions for these pages are currently obtained from Uniprot (14) annotations with plans to expand them into Pfam (15) families in the near future. All the numbers are then linkable to the full detailed information to enable viewing and sorting family members, their bioactive compounds or their 3D structures (Figure 4).

## canSAR-3D

**Figure 5.**
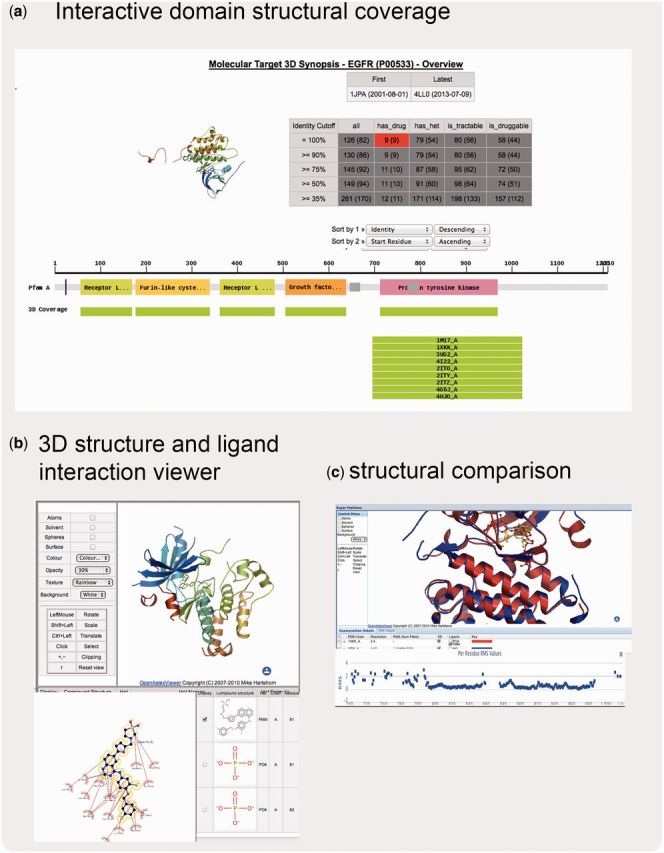
Snapshots of some components of canSAR-3D. (**a**) The 3D explorer shows all structures available for a target (in this case EGFR) and allows filtering based on the availability of bound drugs (as shown) or other ligands, and on structure-based druggability. (**b**) Ligand interaction maps, 3D structural inspection and (**c**) superpositions viewers are available and all are linked seamlessly to chemical bioactivity and protein, genetic and functional data.

3D structure is a powerful tool in understanding the molecular mechanisms of disease and the design and development of new drugs. As well as having access to the large number of protein structures (>93 000) in the Protein Data Bank (PDB) (7), it is important to know how the different structures compare, what domains within the protein are structurally characterized, what ligands or drugs they bind and where druggable pockets potentially exist. canSAR-3D, the 3D structural component of canSAR, contains annotated PDB structures where each structure is linked to the protein and full genomic information, on the one hand, and the full chemical information for its ligand on the other (Figure 5). We have curated the ligands in the PDB into five categories to better distinguish those that are genuine small molecule endogenous ligands or chemical modulators of protein function from biologically unimportant small molecules such as surfactants. The categorization of small molecule ligands is computationally assigned based on the presence of >6 non-Hydrogen atoms. Exceptions to this rule and the surfactants in this list are further classified by manual curation. There is a per-protein structural summary page that graphically represents the structures available for a protein and regions they cover, together with structures of homologues depending on similarity criteria set by the user (Figure 5a).

The full structural complement for each protein is displayed in an interactive browser that allows comparison of the structures and viewing of ligand-interaction maps (Figure 5b, c). 3D structure superposition may also be carried out on-the-fly. The 3D-druggability scores (12,16) for all identified pockets within each structure are automatically calculated and updated in canSAR on a weekly basis. For some families of special interest, we are introducing additional curation and analyses. For example, protein kinase structures are tagged with the activation state of the structure (Figure 5).

## USING canSAR

### Single portal for multidisciplinary data

Users often utilize canSAR as a single-point portal to access broad data available for a gene, protein, drug or cell line. The target, compound and cell line synopses are typical access points for these. More complex use cases typically performed in canSAR include the following examples.

### Identifying tools for probing a target's activity

For a gene or protein of interest, the user can identify available evidence for disease association through altered mutation or expression in patient tissue on the target synopsis. In this same page, the user can identify which cancer cell lines will be useful to use as model systems to study this target, and what are the genetic states of these cellular models. Furthermore, using both the target synopsis and bioactivity exploration, or the cell line synopses, the user can identify which drugs have shown activity against their target or their model cell lines.

### Prioritizing lists of genes for further experiments

For a list of genes from a functional RNAi screen, their proteins can be assessed for ‘druggability’ based on up-to-date structural and chemical information to identify which are the most druggable targets and also what chemical probes have been published for them. These can be investigated individually through the target synopses or in batches of up to 500 genes using the Cancer Protein Annotation Tool in canSAR. This tool provides the user with a spreadsheet summarizing the evidence for druggability, and the availability of structural and chemical probes.

### What is known about a cell line of interest

Using the cell line synopsis, users can explore the genetic and transcriptional make up of a cell line. The user can also identify other cell lines with shared mutations as well as examine drug sensitivity profiles and identify which drugs a cell is sensitive or resistant to.

### Multidisciplinary network analysis

For a target of interest, the user can utilize the protein interaction network to identify druggable targets within the immediate network of the protein of interest. Also, in the same place, the users can identify whether these network neighbors have been reported as hits in a cancer RNAi screen or whether they are mutated in cancer.

## CONCLUDING REMARKS AND FUTURE DEVELOPMENT

Since its initial release, canSAR has grown both in content and functionality. Through the integrated data in canSAR, users can rapidly answer complex scientific questions as exemplified above. Furthermore, examples of using canSAR in this way to identify novel druggable cancer targets have recently been described (16). These and some further examples and use cases are published on the canSAR online documentation pages (http://cansar.icr.ac.uk/cansar/documentation/) and we will be adding to them over time.

Future plans for canSAR include further data growth, especially around the annotation of patient-derived experimental data, cancer clinical trial information, increased integration with the IMEx consortium (2) for the annotation of protein-network data and introducing tissue imaging data. Furthermore, a number of additional expert tools are planned including the introduction of Kaplan–Meier plot tools to identify correlation between underlying genetics and patient, mutation impact summaries and pathway exploration tools—all of which have been requested by our users. We will also develop interactive gene/protein visualization tools, specific cancer disease browsing and cancer clinical trial navigation tools in addition to further enhancements and functionality in response to feedback from our users.

## FUNDING

This work is funded though Cancer Research UK core funding to the Cancer Therapeutics Unit grant number [C309/A11566]. Funding for open access charge: [C309/A11566].

*Conflict of interest statement*. The authors are employees of The Institute of Cancer Research, which has a commercial interest in the discovery and development of anticancer drugs, and operates a rewards to inventors scheme. B.A.L. is a former employee of Inpharmatica Ltd.
